# HIV-1 Tat Protein Increases Microglial Outward K^+^ Current and Resultant Neurotoxic Activity

**DOI:** 10.1371/journal.pone.0064904

**Published:** 2013-05-30

**Authors:** Jianuo Liu, Peng Xu, Cory Collins, Han Liu, Jingdong Zhang, James P. Keblesh, Huangui Xiong

**Affiliations:** 1 Neurophysiology Laboratory, Department of Pharmacology and Experimental Neuroscience, University of Nebraska Medical Center, Omaha, Nebraska, United States of America; 2 Department of Pathology and Microbiology, University of Nebraska Medical Center, Omaha, Nebraska, United States of America; Virginia Commonwealth University, United States of America

## Abstract

Microglia plays a crucial role in the pathogenesis of HIV-1-associated neurocognitive disorders. Increasing evidence indicates the voltage-gated potassium (K_v_) channels are involved in the regulation of microglia function, prompting us to hypothesize K_v_ channels may also be involved in microglia-mediated neurotoxic activity in HIV-1-infected brain. To test this hypothesis, we investigated the involvement of K_v_ channels in the response of microglia to HIV-1 Tat protein. Treatment of rat microglia with HIV-1 Tat protein (200 ng/ml) resulted in pro-inflammatory microglial activation, as indicated by increases in TNF-α, IL-1β, reactive oxygen species, and nitric oxide, which were accompanied by enhanced outward K^+^ current and K_v_1.3 channel expression. Suppression of microglial K_v_1.3 channel activity, either with K_v_1.3 channel blockers Margatoxin, 5-(4-Phenoxybutoxy)psoralen, or broad-spectrum K^+^ channel blocker 4-Aminopyridine, or by knockdown of K_v_1.3 expression via transfection of microglia with K_v_1.3 siRNA, was found to abrogate the neurotoxic activity of microglia resulting from HIV-1 Tat exposure. Furthermore, HIV-1 Tat-induced neuronal apoptosis was attenuated with the application of supernatant collected from K^+^ channel blocker-treated microglia. Lastly, the intracellular signaling pathways associated with K_v_1.3 were investigated and enhancement of microglial K_v_1.3 was found to correspond with an increase in Erk1/2 mitogen-activated protein kinase activation. These data suggest targeting microglial K_v_1.3 channels may be a potential new avenue of therapy for inflammation-mediated neurological disorders.

## Introduction

Individuals infected with human immunodeficiency virus type 1 (HIV-1) often suffer from neurocognitive impairments which are referred to as HIV-1-associated neurocognitive disorders (HAND) [1], [2]. The severity of HAND varies, ranging from asymptomatic neurocognitive impairment to its severest form: HIV-1-associated dementia [2]. Despite the widespread use of potent antiretroviral therapy (ART), the incidence of HAND has not been fully prevented and its prevalence remains high ranging from 39% to 52% in varied settings [3], [4], [5]. Although the persistence of HAND is multifactorial, the paucity of effective therapeutic modalities in the control of brain macrophage and microglia activation and resultant production of neurotoxins, a striking pathological feature in HIV-1-infected brain, plays an important role as pathogenesis and severity of HAND is highly correlated with activated brain macrophages and microglia but not the presence and amount of virus in the brain [6], [7]. It is well known that the activated microglia secrete a number of neurotoxins including, but not limited to, pro-inflammatory cytokines, and excitatory amino acids, reactive oxygen species (ROS), nitric oxygen (NO), which can result in neuronal injury and consequent neurocognitive impairments [8], [9], [10]. As such, studies on elucidation of the mechanisms by which HIV-1 triggers microglial neurotoxicity and identification of specific target(s) to control microglia activation are imperative.

Voltage-gated potassium (K_v_) channels have recently gained much attention as the potential targets for therapy of neurological disorders [11], [12]. Electrophysiological studies of microglia in culture and tissue slices have demonstrated that microglia express several types of K_v_ channels including inward rectifier K_ir_2.1 and outward rectifiers K_v_1.5 and K_v_1.3. Exposure to a variety of activating stimuli produces a characteristic pattern of up-regulation of K_v_1.3 [13], [14], [15], [16]. Whereas the expression of K_ir_2.1 channels are often found in resting microglia [17], [18], the expression of K_v_1.5 and K_v_1.3, especially the latter, appear to be associated with microglia activation and neurotoxin production [15], [19], [20], [21]. Indeed, studies have shown that activation of microglia results in neuronal injury through a process requiring K_v_1.3 activity in microglia. Studies have also shown that blocking microglia K_v_1.3 or decrease of K_v_1.3 expression inhibits microglia-induced neurotoxicity [22], [23]. We hypothesize that HIV-1 brain infection triggers microglia neurotoxic activity by increasing K_v_1.3 activity, resulting in microglia activation and consequent neuronal injury. To test this hypothesis, we studied involvement of K_v_1.3 in HIV-1 Tat protein-induced microglia activation and resultant neurotoxic activity in primary microglia culture prepared from Sprague-Dawley rats. Our results demonstrated that HIV-1 Tat increases microglia production of neurotoxins and resultant neurotoxicity through enhancements of K_v_1.3 protein expression and outward K^+^ currents, which can be blocked by pretreatment of microglia with specific K_v_ channel blockers Margatoxin (MgTx) or 5-(4-Phenoxybutoxy)psoralen (PAP), or by transfection of microglia with K_v_1.3 siRNA, suggesting an involvement of K_v_1.3 in microglia-mediated neurotoxic activity. The enhancements of K_v_1.3 channel activity and microglia neurotoxicity resulting from HIV-1 Tat protein exposure are dependent on the Erk1/2 MAPK signal pathway. Here we present evidence for the reduction of neurotoxic secretions from microglia and associated neuronal injury by modulation of K^+^ channel activity as a potential new treatment approach deserving further investigation.

## Materials and Methods

### Animals

Sprague-Dawley rats were purchased from Charles River Laboratories (Wilmington, MA) and maintained under ethical guidelines for care of laboratory animals at the University of Nebraska Medical Center. All animal-use procedures were reviewed and approved by the Institutional Animal Care and Use Committee (IACUC) of University of Nebraska Medical Center (IACUC # 00-062-07).

### Primary microglia and neuron cultures

Microglia were derived from the cerebral cortices of 0–1 day old neonatal Sprague Dawley rats as described previously [24]. Cortical tissues were dissected in cold Hanks' Balanced Salt Solution (HBSS: Madiatech, Inc. Manassas, VA) and digested in a solution consisting of 0.25% trypsin and 200 Kunitz DNase (Sigma, St. Louis. MO) at 37°C for 30 min. Tissues were then suspended in cold HBSS and filtered using 100 µm and 40 µm cellular strainers (BD Bioscience, Durham, NC). Isolated cells (30×10^6^) were plated into T75 cm^2^ flasks in a high-glucose Dulbecco's modified Eagle's medium (DMEM) supplemented with 10% fetal bovine serum (FBS), 2 mM L-glutamine, 1% penicillin/streptomycin, and 1 µg/ml macrophage colony-stimulating factor (Life Technologies, Grand Island, NY). After 10 day's culture, flasks were shaken gently to detach cells, which were plated based on experimental requirements in either 35 mm^2^ culture dishes (2.5×10^6^ cells/dish), 60 mm^2^ culture dishes (7.5×10^6^ cells/dish), 12-well plates (1×10^6^/well), or 96-well plates (0.4×10^6^/well) and incubated at 37°C. After 30 min, suspended glial cells were removed by aspiration of culture supernatant and fresh culture media was applied. The resulting cultures were stained with OX-42 antibody (Serotec, Oxford, UK), a microglial CR3/CD11b receptor marker, and determined to consist of 98–100% microglia.

Primary cortical neurons were prepared from 18-day old Sprague Dawley embryonic rats (Charles River Laboratories). Dissected cortices were digested with 0.25% trypsin and DNase (200 Kunitz) in 37°C for 15 min, then filtered through 100 and 40 µm pore cellular strainers. Isolates were seeded in pre-coated poly-D-lysine plates at a density of 0.05×10^6^ cells/well in 96-well plates, 0.15×10^6^ cells/well in 24-well plates, or 1.0×10^6^ cells/well in 6 well-plates. Neuronal cultures were maintained at 37°C for 10 days in neurobasal medium (Gibco by Life Technologies) supplemented with 2% B27, 1% penicillin/streptomycin and 0.5 mM L-glutamine (Invitrogen by Life Technologies). The purity of neuronal cells was determined to be >90% by staining with microtubule-associated protein-2 antibody (MAP-2: 1∶1000, Chemicon International, Inc. Temecula, California).

### Electrophysiology

Whole-cell outward K^+^ currents were recorded from primary rat microglia cultures at room temperature. Microglia were perfused with artificial cerebrospinal fluid (ACSF) contained (in mM) NaCl 150, KCl 4.5, CaCl_2_ 2, MgCl_2_ 1, HEPES 5, and glucose 11. The ACSF was continuously oxygenated with 95% O_2_ and 5% CO_2_ with a pH of 7.4 and an osmolarity of 310 mOsm. Patch-clamp electrodes were made from borosilicate glass capillaries (WPI, Sarasota, FL) with a resistance of 4–6 Ω when filled with pipette solution contained (in mM) KCl 150, MgCl_2_ 1, CaCl_2_ 1, EGTA 11, and HEPES 10; adjusted to a pH of 7.3 with KOH. Voltage-dependent currents were evoked by voltage steps (600 ms in duration) with the first step from the holding potential of −70 mV to −170 mV and then stepped to +50 mV with a 20 mV increments [13]. The seal resistance was 1–10 GΩ. Junction potentials were corrected and the cell capacitance was compensated (∼70%) in most cells. Current signals were amplified with an Axopatch 200B amplifier (Molecular Devices, Sunnyvale, CA). The current traces were displayed and recorded on a Dell computer using a pClamp 10.1 data acquisition/analysis system. K^+^ current density (pA/pF) was calculated by dividing the peak current amplitude generated at a given voltage step by the cell capacitance.

### Measurement of reactive oxygen species (ROS) and Nitric oxide (NO) production

Intracellular ROS were measured by fluorometric assay using 2′, 7′-dichlorofluorescein diacetate (DCFH-DA, Sigma, St. Louis, MO), a well-established compound for detecting and quantifying intracellular ROS production. Microglia were first treated for 30 minutes with a K_V_1.3 channel blocker, either 5 nM MgTx, 10 nM PAP, or 1 mM 4-AP (all purchased from Sigma-Aldrich Co, LLC, St. Louis, MO), followed by treatment with either 200 ng/ml of HIV-1 Tat_1-72_ protein (Tat) or heat inactivated- Tat_1-72_ (HI Tat) (purchased from University of Kentucky). After 24 hr, microglia were exposed to 20 µM DCFH-DA for 30 min. Cells were then washed twice in PBS and the fluorescence immediately measured in a plate reader at an excitation wavelength of 485 nm and an emission wavelength of 520 nm.

NO production was estimated by measuring the concentration of nitrite using the Griess Reagent System according to the manufacturer instruction (Promega, Madison, WI). 50 µl aliquots of supernatant were collected from cultures of pre-treated microglia, mixed with equal volume of Sulfanilamide Solution for 10 min, combined with 50 µl of NED solution, and incubated for 30 min at room temperature. The optical density was then measured at 520 nm and 540 nm using an ELISA plate reader. All experiments were repeated at least three times.

### Cytokine assay

Cytokines IL-1β and TNF-α in pre-treated microglia supernatants were quantified using specific enzyme-linked immunosorbent assay (ELISA) kits (R&D Systems) in accordance with manufacturer protocol.

### TUNEL staining and MTT assay

Neuronal apoptosis was evaluated using a Fluorescein *In Situ* Cell Death Detection Kit (Roche Applied Science, Indianapolis, IN). In brief, neurons growing on poly-D-lysine-coated coverslips (0.15×10^6^ cells/well in a 24-well plate) were exposed to supernatants collected from pre-treated microglia at 1∶5 dilution for 24 hr. Neurons were then fixed with 4% paraformaldehyde (PFA) and permeabilized with 0.1% Triton X-100. Neurons were subsequently incubated in the TUNEL reaction mixture for 1 hr at 37°C and then mounted using ProLong Gold antifade reagent with 4′,6′-diamidino-2-phenylindol (DAPI) counterstain (Molecular Probes, Eugene, OR). Cells were visualized using the 40× oil-immersion objective of a Zeiss LSM 510 META NLO microscope (Zeiss MicroImaging, Inc., Thornwood, NY). The percentage of apoptotic neurons was determined based on TUNEL-positive cells normalized to DAPI-stained nuclei.

Cell viability was assessed by MTT assay. Pre-treated neurons were exposed to fresh neurobasal medium containing 500 µg/ml 3-(4,5-dimethylthiazol-2-yl)-2,5-diphenyl tetrazolium bromide (MTT) for 3 hr. The MTT solution was then replaced with 300 µl of dimethyl sphingosine (DMSO: Sigma-Aldrich) for cell lysis and the optical density (OD) was measured at 560 nm.

### Immunocytochemistry

Microglia were seeded on coverslips at a density of 1.0×10^6^/well in 12-well plates, treated for 30 min with 5 nM MgTx, 10 nM PAP, or 1 mM 4-AP, and incubated with Tat (200 ng/ml). After 24 hr, cells were washed, fixed with 4% PFA for 30 min, and incubated with 10% normal goat serum blocking solution for 30 min. Primary antibodies (Ab) anti-CD11b Ab (CD11b; 1∶500; abcam, Cambridge, MA) and anti-K_V_1.3 (KCNA3 1∶100, Alomone Lab Ltd, Jerusalem Israel) were then applied to coverslips for 3 hr at RT. Cells were subsequently incubated for 1 hr with Alexa Fluor 488 and Alexa Fluor 594-conjugated secondary Abs (1∶1000, Molecular Probes, Invitrogen by Life Technologies). After washing, cells were mounted using ProLong Gold antifade reagent with DAPI counterstain (Molecular Probes). Images were obtained using the 40× oil-immersion objective of a Zeiss LSM 510 META NLO microscope. A minimum of 5 images were taken from each slide.

### Immunohistochemistry

Brain hippocampal tissues were dissected out from 20–30 d old Sprague Dawley rats (Charles River Laboratories), cut into slices at 400 µM in thickness, and placed on a 100 µm pore cellular strainer in a 6-well plate. The hippocampal slices were then treated for 30 min with MgTx (5 nM), PAP (10 nM), or 4-AP (1 mM) and subsequently incubated with 200 ng/ml of Tat. After 24 hr, hippocampal slices were fixed in 4% PFA for another 24 hr, immersed in 30% sucrose for 48 hr, embedded in optimal cutting temperature (OCT) media, and cryosectioned to a thickness of 10 µm. Hippocampal sections were then immunostained with either anti-Iba1 Ab (1∶500, WAKO Chemicals USA, Inc. Richmond, VA), K_V_1.3 Ab (1∶200, Santa Cruz biotechnology, Inc, CA), or TUNEL stain and visualized using the 40× oil-immersion objective of a Zeiss LSM 510 META NLO microscope. A minimum of 5 images were taken from each slides.

### Western blot analysis

Membrane proteins were prepared using a Membrane Protein Extraction Kit (BioVision, Mountain View, CA, USA) according to manufacturer instruction, while total proteins were isolated using a RIPA buffer (Bio-Rad, Hercules, CA). Well volumes of 20 µg for membrane proteins and 30 µg for total proteins were separated by electrophoresis using 4–15% Mini-PROTEAN TGX precast gel and transferred to nitrocellulose polyvinylidene difluoride (PVDF) membranes. PVDF membranes were then blocked with 5% dry milk in Tris-Buffered Saline (TBS) (all products from Bio-Rad Laboratories, Hercules, CA) and probed overnight at 4°C with either rabbit polyclonal K_V_1.3 (1∶100; Alomone Lab, Israel), phospho-p44/42 MAPK (pERK1/2), total p44/42 MAPK (ERK1/2) (1∶1000; Cell Signaling Technology, Danvers, MA), or anti-mouse β-actin monoclonal antibody (1∶10,000, Sigma-Aldrich) primary Abs. Membranes were next washed (4×10 min) in TBS with 0.2% Tween (TBS-T) and incubated for 1 hr at RT with either horseradish peroxidase (HRP)-conjugated anti-rabbit or anti-mouse secondary antibody (1∶10,000, Jackson ImmunoResearch Laboratories, West Grove, PA). Labeled proteins were visualized by Pierce ECL Western Blotting Substrate (Thermo Scientific, Rockford, IL). Band densities of p-pErk1/2 were normalized to total ERK1/2 in each sample.

### Reverse transcription (RT)-PCR

Total RNA was isolated from microglia using TRIzol Reagent (Invitrogen, Carlsbad, CA), purified by RNeasy Mini Kit (QIAGEN, Inc., Valencia, CA), and reverse transcribed at 65°C for 50 min according to SuperScript III reverse transcriptase (Invitrogen) protocol. PCR amplification with Platinum PCR SuperMix (Invitrogen) then included 2 min incubation at 94°C followed by 30 cycles consisting of a 30 s denaturing phase at 94°C, a 30 s annealing phase at 55°C, a 1 min extension phase at 72°C, and a final extension phase of 10 min at 72°C. Densitometry analysis of DNA products was performed using Northern Eclipse 6.0 software (Bio-Rad) and results normalized to β-actin internal controls. PCR primers used were as follows: forward K_v_1.3 primer was GTA CTT CGA CCC GCT CCG CAA TGA; reverse K_v_1.3 primer was GGG CAA GCA AAG AAT CGC ACC AG; forward β-Actin primer was GTG GGG CGC CCC AGG CAC CA; reverse β-Actin primer was CTT CCT TAA TGT CAC GCA CGA TTT C.

### siRNA transfection

Pre-designed ON-TARGETplus SMARTpool siRNA against rat KCNA3 (K_v_1.3, NM-019270) mRNA was purchased from Dharmacon, Inc. (Chicago, IL). Microglia plated to 2×10^6^ cells/well in 6-well plates were transfected with 100 µl of 2 µM siRNA for 48 or 72 hr in the presence of Dharma FECT Transfection Reagent (Dharmacon, Inc) according to the manufacturer instruction. A non-specific ON-TARGETplus GAPD Control Pool siRNA (rat) (Dharmacon, Inc) was also similarly transfected at the same concentration as the control. Transfected microglia were then incubated for 24 hr with or without Tat (200 ng/ml), after which the supernatant was collected (for conditioned media) and the cells were harvested (for preparation of RNA and protein).

### Statistical Analysis

Experimental data are expressed as mean±S.D. unless otherwise indicated. Statistical analyses were performed by Student *t* tests. A minimum *p* value of 0.05 was estimated as the significance level for all tests.

## Results

### HIV-1 Tat exposure induces K_v_1.3 currents in microglia

**Figure 1:**
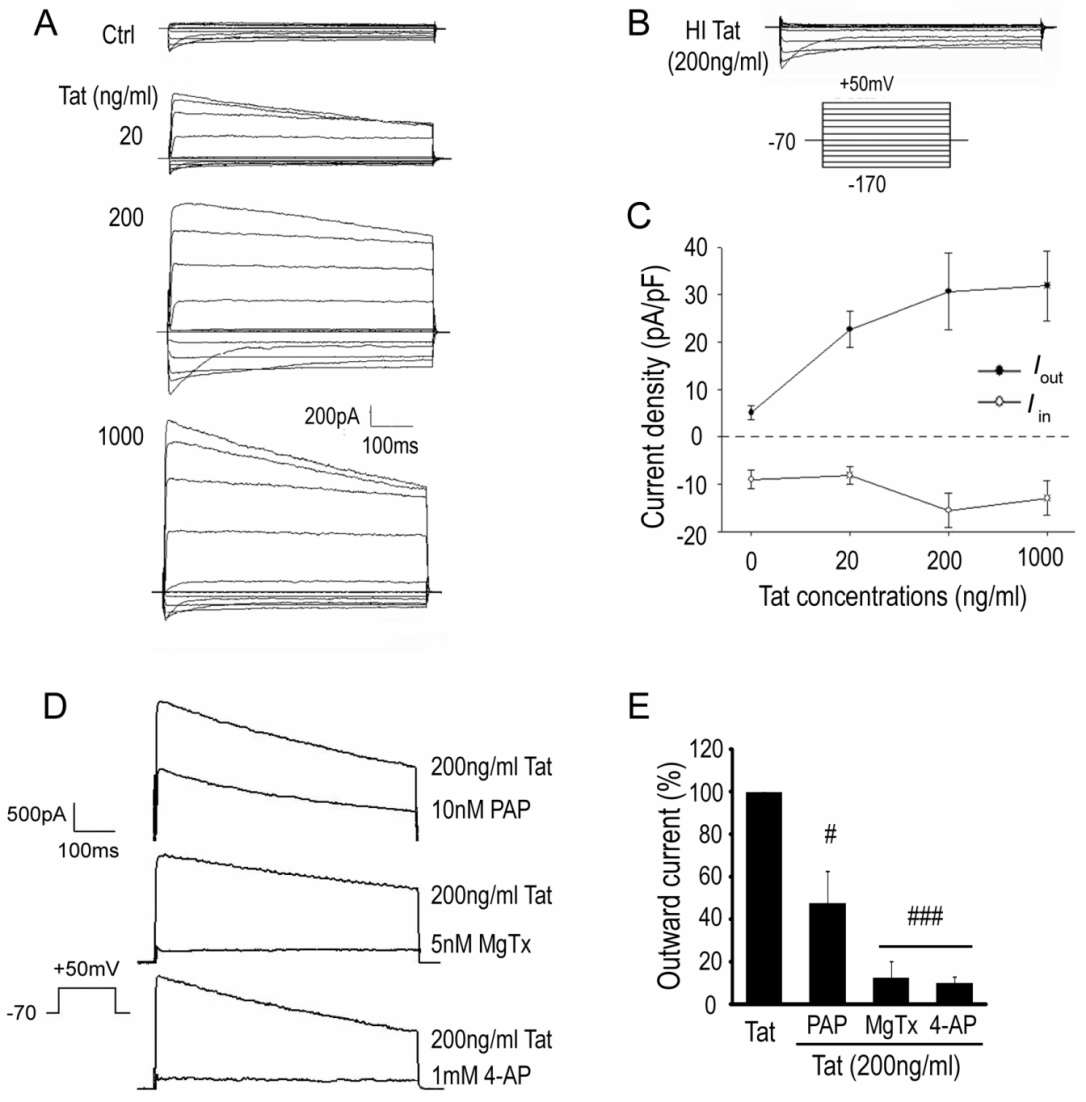
HIV-1 Tat protein enhances outward K^+^ currents (*I*_out_) in rat microglia. **A**: Representative whole-cell membrane currents recorded from microglia treated with or without Tat at varied concentrations (0, 20, 200, 1000 ng/ml). **B**: Whole-cell membrane current recordings of microglia treated with heat-inactivated Tat (200 ng/ml). **C**: Dose-response curve of the Tat-induced effect on *I*_out_. Current densities in response to different concentrations of Tat is calculated (mean ± S.E.M) from 32, 25, 27 and 24 different cells in control, 20 ng/ml, 200 ng/ml, 1000 ng/ml groups. **D**: Pharmacology of Tat-induced *I*_out_. Tat induced *I*_out_ were evoked by voltage from the holding potential −70 mV to +50 mV for 600 ms are shown before and during superfusion with extracellular solution containing 10 nM PAP, 5 nM MgTX, 1 mM 4-AP. **E**: Blockade of Tat(200 ng/ml) enhancement of *I*_out_ by specific Kv1.3 blockers PAP (n = 8) and MgTx (n = 8) or by a broad spectrum K^+^ channel blocker 4-AP (n = 7). # p<0.05 vs Tat; ### p<0.001 vs Tat.

HIV-1 pathogenesis involves the release of soluble viral proteins such as gp120, Tat, and Nef. In previous studies, we demonstrated HIV-1 gp120 IIIB enhanced whole-cell outward K^+^ current in cultured rat microglia through K_v_1.3 channels [24], [25]. Here we propose HIV-1 Tat protein may alter microglia channel profiles in a similar manner. To test our hypothesis, we first examined the effect of HIV-1 Tat protein on the electrophysiological properties of microglia. Although sera levels of HIV-1 Tat have been reported to range from 1–40 ng/ml in HIV-1 positive individuals [26], [27], localized concentrations are reasoned to be higher and nM concentrations are commonly used *in vitro* to elicit the effects of Tat exposure [28], [29]. In our study, purified rat microglia were pretreated with HIV-1 Tat protein at 20–1000 ng/ml for 24 hr before recording. Electrophysiological recordings were performed using a conventional whole-cell recording under voltage clamp configuration. The average inward K^+^ current (*I*_in_) and outward K^+^ current (*I*_out_) densities (pA/pF) were calculated by dividing the K^+^ current amplitude by the membrane capacitance. At hyperpolarizing potentials, both untreated and Tat treated microglia displayed an *I*_in_ (Fig. 1A). The *I*_in_ density in untreated microglia (−8.96±3.76 pA/pF; n = 32) was only minimally affected by exposure to either 20 ng/ml Tat (−8.14±3.61 pA/pF; n = 25), 200 ng/ml Tat (−15.5±7.36 pA/pF; n = 27), or 1000 ng/ml Tat (−12.9±7.21 pA/pF; n = 24). With depolarizing pulses however, Tat treated microglia responded with a substantial *I*_out_ (Fig. 1A). In fact, the *I*_out_ density in microglia pretreated with 20 ng/ml Tat (22.69±7.46 pA/pF; n = 25) was over fourfold greater than in untreated microglia (5.09±2.84 pA/pF; n = 32). Furthermore, the *I*_out_ densities in microglia exposed to 200 ng/ml Tat (30.7±16.29 pA/pF; n = 27) and 1000 ng/ml Tat (31.86±14.69 pA/pF; n = 24) demonstrate this effect to be dose-dependent (Fig. 1C). To confirm these observations were due specifically to Tat protein function, we disrupted its tertiary structure with heat (75°C for 5 hr) prior to incubation with microglia. Similarly to untreated cells, microglia treated with 200 ng/ml heat-inactivated Tat protein (HI Tat, n = 9) exhibited hyperpolarization-evoked *I*_in_ currents and lacked significant *I*_out_ current in response to depolarizing pulses (Fig. 1B). Next, to determine whether the Tat-induced *I*_out_ currents were conducted via K_v_1.3 channels, Tat-treated microglia were perfused with ACSF contained specific K_v_1.3 blockers PAP (10 nM), MgTx (5 nM), or a broad spectrum K_v_ channel blocker 4-AP (1 mM), and the *I*_out_ was significantly reduced by 52.1±14.72% (n = 8), 87.26±7.79% (n = 8) or 89.81±3.09% (n = 7) (Fig. 1D & 1E). Taken together, these findings strongly suggest HIV-1 Tat exposure induces outward K^+^ currents in microglia through K_v_1.3 channels.

### HIV-1 Tat upregulates K_V_ 1.3 expression in rat microglia

**Figure 2:**
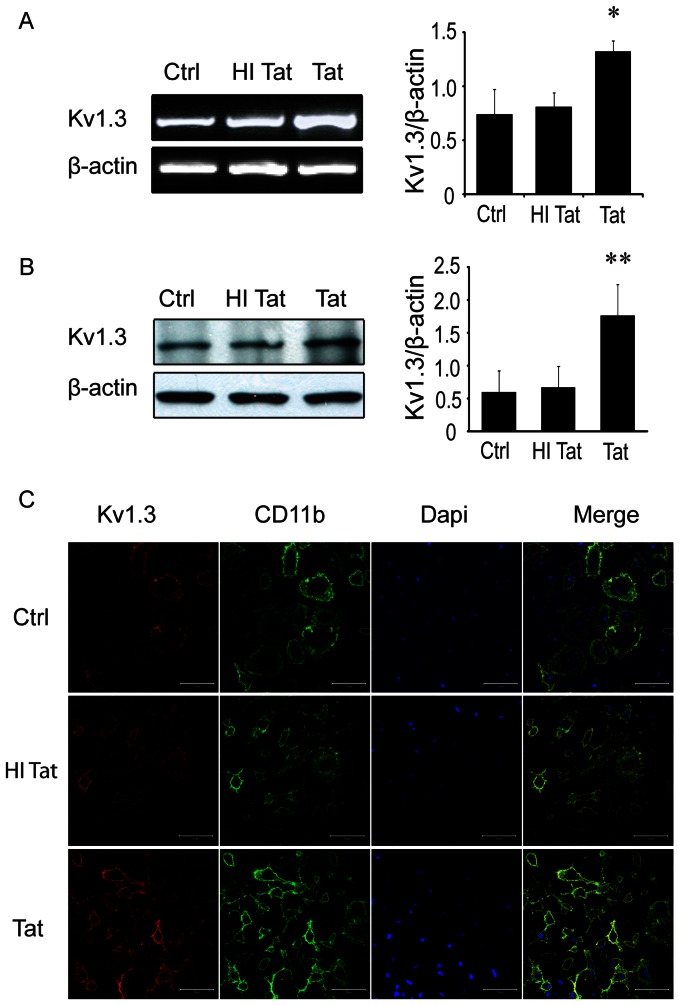
Tat upregulates microglia K_v_1.3 channel expression. **A**: A representative RT-PCR gel (left) and its corresponding densitometry bar graph (right) showing enhanced levels of K_V_1.3 mRNA in microglia treated with Tat (200 ng/ml), but not heat-inactivated Tat (HI Tat, 200 ng/ml). **B**: The levels of K_v_1.3 protein was also elevated by Tat as detected by Western blot (left) and its densitometry bar graph (right). Data were obtained from three independent experiments. **C**: Tat-activated microglia were immunostained for expression of K_v_1.3 (red), CD11b (green) and Dapi nuclei. Images were visualized by fluorescent confocal microscopy at ×400 original magnification. Scale bars are equal to 50 µm. * *p*<0.05, ** *p*<0.01 vs Ctrl.

K_v_ channel activity can be altered by numerous factors, including by membrane potential, redox potential, transcription, translation, posttranslational modification, or via direct interaction with organic molecules or peptides. To better determine the mechanism through which Tat induces K_v_1.3currents in rat microglia, K_v_1.3 mRNA and protein levels were ascertained by RT-PCR and western blot. RT-PCR performed after 24 hr incubation of rat microglia with 200 ng/ml Tat protein showed marked elevation in K_v_1.3 mRNA expression (Fig. 2A), with K_v_1.3 mRNA density in Tat-treated cells (1.32±0.10) measuring 1.8 times greater than in untreated microglia (0.74±023). As a negative control, the K_v_1.3 mRNA density was measured in microglia treated with 200 ng/ml heat-inactivated Tat protein and found to be essentially unchanged (0.81±0.13) (Fig. 2A). Consistent with these effects, treatment of microglia with 200 ng/ml Tat for 24 hr led to a nearly threefold increase in K_v_1.3 protein levels (Fig. 2B), which was further confirmed and visualized by immunocytochemical labeling (Fig. 2C). These findings clearly indicate Tat protein exposure upregulates the expression of K_v_1.3 channels in rat microglia.

### Involvement of K_V_1.3 in Tat-induced microglia-mediated neurotoxicity

**Figure 3:**
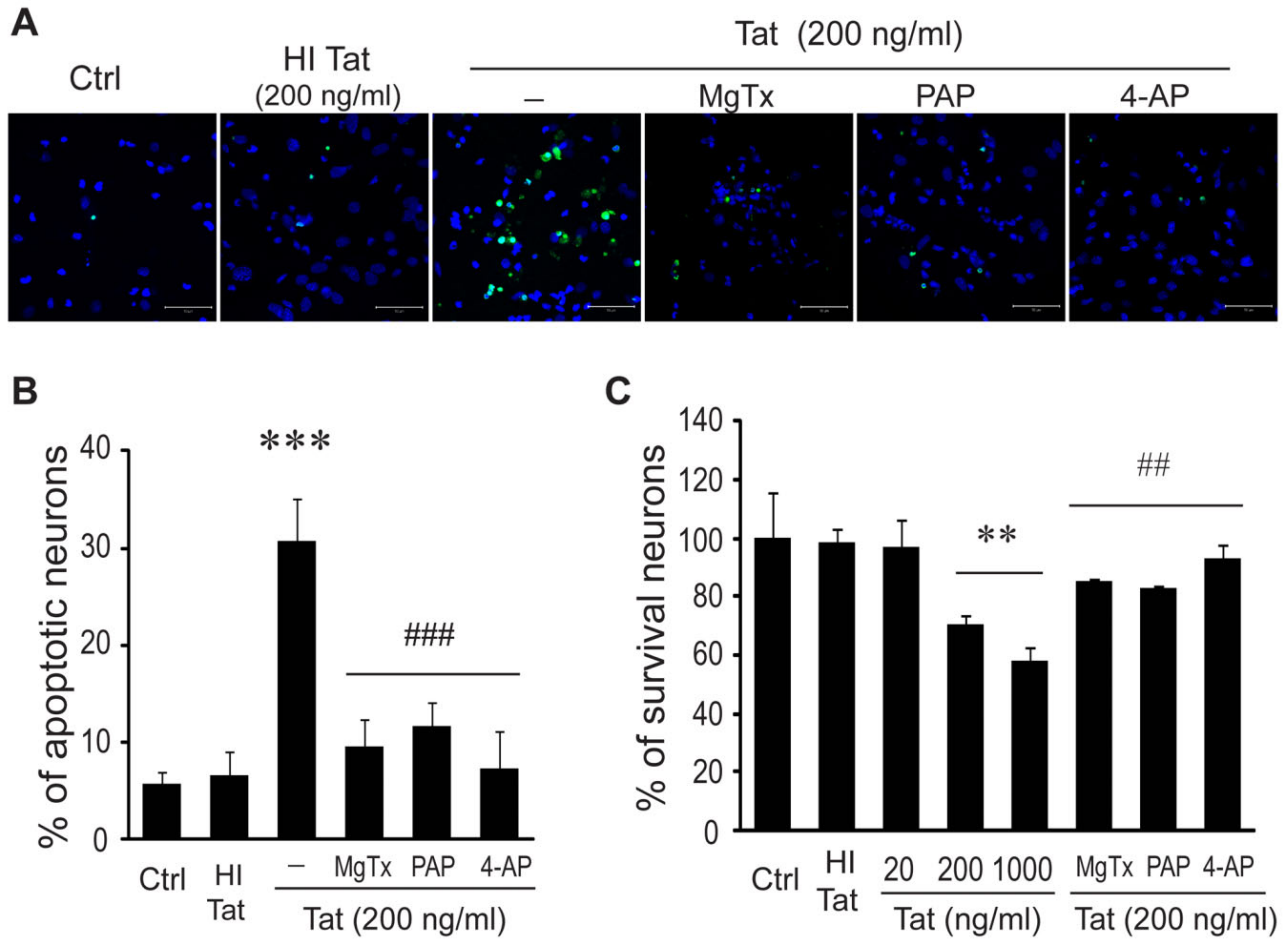
K_v_ channel antagonists attenuate Tat-induced microglia neurotoxicity. Neurons were exposed to conditioned media recovered from microglia pre-treated with MgTx (5 nM), PAP (10 nM), or 4-AP (1 mM) for 30 min followed by Tat at varied concentrations (0, 20, 200, 1000 ng/ml). After 24 hr treatment, TUNEL staining and MTT were performed. **A**: TUNEL positive neurons were visualized by confocal microscopy at ×400 original magnification. Scale bar equals 50 µm. Note that Tat-treated conditioned media, but not heat inactivated Tat (HI Tat)-treated conditioned media, induced neuronal apoptosis and that the Tat-induced neuronal apoptosis was blocked by MgTx, PAP or 4-AP. **B**: Quantitative exhibition of apoptotic neurons determined by ratio of the number of TUNEL-positive cells to the total number of Dapi-positive cells under different experimental conditions as indicated. **C**: MTT assay showed increased viabilities in MgTx, PAP, and 4-AP-treated groups, respectively. Data were from three independent experiments. ** p<0.01, *** p<0.001 vs Ctrl; ^##^ p<0.01, ^###^ p<0.001 vs conditioned media treated with Tat alone.

Having established Tat protein exposure increases K_v_1.3 expression and current density in microglia, we next sought to determine if this change in channel profile contributes to the neurotoxicity of HIV-1 Tat-activated microglia. Microglial supernatants were first collected after 24 hr treatment with either HIV-1 Tat protein (at doses of 0, 20, 200, and 1000 ng/ml) or heat-inactivated Tat (200 ng/ml). Rat cortical neurons growing on poly-D-lysine-coated coverslips in 24-well plates were then subjected to these supernatants (1∶5 dilution) for an additional 24 hr and neuronal viability was assessed by MTT assay. As shown in Figure 3C, neuronal cell viability was found to be essentially unaffected at doses of 0 ng/ml (100%) and 20 ng/ml (97.02±9.38%), however was progressively and significantly reduced as the microglial supernatant Tat treatment dose was increased to 200 ng/ml (70.19±3.33%; p<.01) and 1000 ng/ml (58.45±4.65%; p<.01). Neuronal viability was further unaffected by incubation with supernatant collected from microglia treated with heat-inactivated Tat protein (HI Tat, 98.4±4.48%), demonstrating the dose-dependent reductions in viability to be specific to functional Tat protein.

Next, the role of K_v_ channels in Tat-activated microglial neurotoxicity was investigated using a similar experimental design and examining the effect of K_V_ channel blocker pretreatment on neuronal health. Microglia were first pretreated with either 5 nM MgTx, 10 nM PAP, or 1 mM 4-AP for 30 min. Based on the capacity to substantially reduce neuronal viability, a dose of 200 ng/ml Tat protein was applied and cultures incubated for 24 hr. Microglia supernatants were then collected and added to cultured rat cortical neurons at a dilution ratio of 1∶5. After 24 hr, neuronal viability and neuronal apoptosis were assessed by MTT assay and TUNEL staining, respectively. As noted previously, cell viability was shown by MTT assay to be decreased in neuronal cultures exposed to Tat-treated microglial supernatant (70.19±3.33%), an effect which was significantly attenuated (p<.01) by the pretreatment of microglia with MgTx (85.46±1.00%), PAP (82.95±0.54%), or 4-AP (92.83±4.66%) (Fig. 3C). The complementary study of neuronal apoptosis revealed similar findings, with the percentage of apoptotic neurons greatly increasing (p<.001) with application of Tat protein (30.68±4.3%) compared to control (5.6±1.3%) (Fig. 3A & 3B). Again, this result was largely reversed (p<.01) when microglial cultures were treated with MgTx (9.56±2.78%), PAP (11.72±2.42%), or 4-AP (7.29±3.84%) prior to the application of Tat protein (Fig. 3A & 3B). The recovery of neuronal viability and attenuation of neuronal apoptosis by K_V_ channel blockade, including the use of K_v_1.3 specific inhibitors, suggests that K_v_1.3 channel activity greatly impacts Tat-induced microglia-mediated neurotoxicity.

### K_V_1.3 channel blockade decreases neurotoxic secretions by Tat-activated microglia

**Figure 4:**
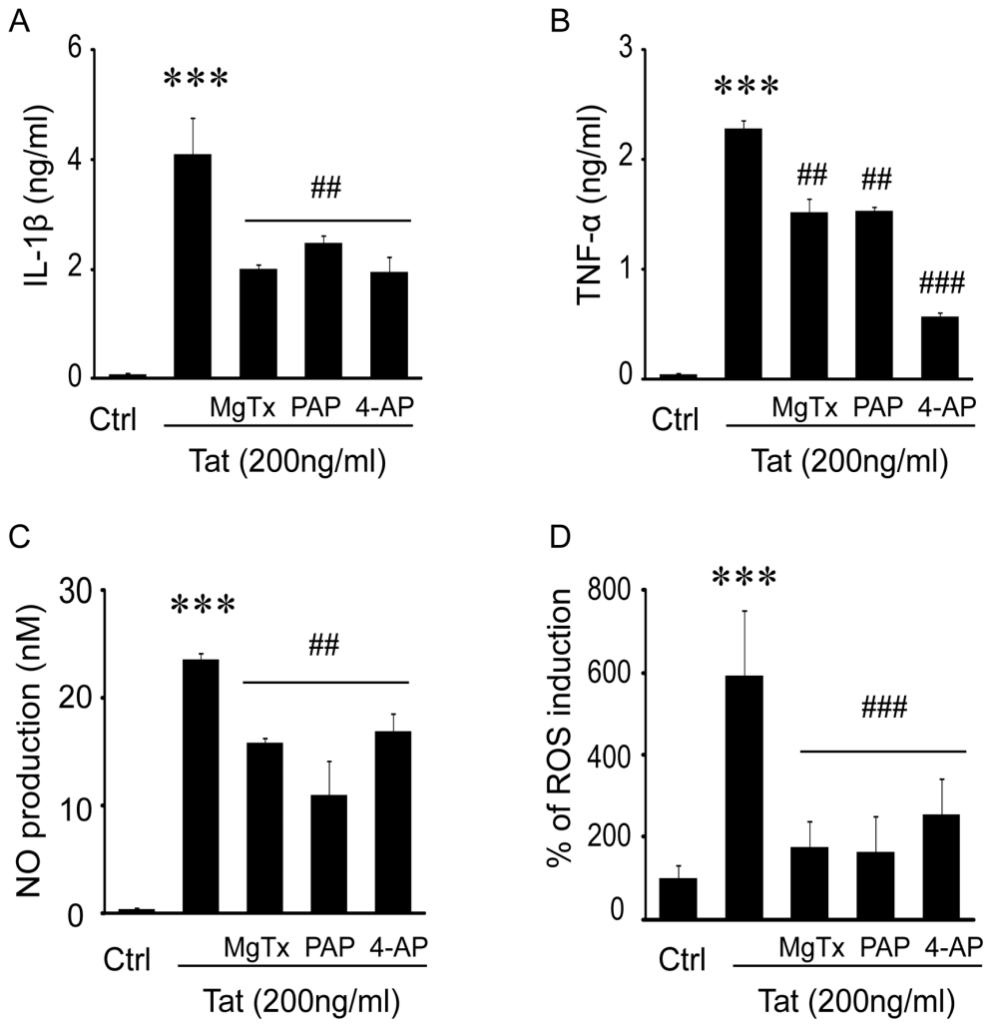
K_v_ channel blockers inhibited Tat-activated microglia secretion of neurotoxins. Microglia were treated with MgTx (5 nM), PAP (10 nM), or 4-AP (1 mM) for 30 min before addition of Tat at 200 ng/ml. After 24 hr incubation, the supernatants were harvested for detection of IL-1β (**A**), TNF-α (**B**), NO (**C**) and the cells were used for analysis of ROS production (**D**). Data presented were from three independent experiments. *** *p*<0.001) vs Ctrl; ^##^ p<0.01 or ^###^ p<0.001 illustrates Tat alone vs pre-Tat plus MgTx, PAP or 4-AP.

The production and release of bioactive molecules by activated microglia is believed to be the principal pathway in HAND associated neuropathology. To better clarify the functional role of K_v_1.3 channels in this process, we next examined the capacity for Tat exposure to induce the secretion of proinflammatory cytokines such as TNF-α and IL-1β, in the presence and absence of K_v_ channel blockers. For this experiment, purified microglia were first pre-treated for 30 min with a K_v_ channel blocker, either MgTx (5 nM), PAP (10 nM), or 4-AP (1 mM), and then incubated with 200 ng/ml Tat protein for 24 hr. Subsequent cytokine assays revealed marked increases (*p*<.001) in levels of TNF-α (2.28±0.07 ng/ml) and IL-1β (4.10±0.68 ng/ml) in the supernatants of Tat-treated microglia compared to the nearly undetectable levels in untreated controls (Fig. 4A & 4B). Further, this Tat-induced production of cytokines was significantly inhibited (*p*<.01) in cultures pretreated with K_v_ channel blockers MgTx, PAP, or 4-AP. In addition to measuring cytokines, we used a similar experimental design to measure other neurotoxic microglial products including NO and ROS. The level of NO in supernatants from Tat-treated microglia was (23.65±0.60 nM) compared to (0.42±0.16 nM) in untreated matched controls (Fig. 4C). This production was significantly limited (*p*<.01) by blockade of K_V_ channels using either MgTx (15.81±0.56 nM), PAP (11.00±3.20 nM), or 4-AP (16.96±1.60 nM). Similarly, ROS production in Tat-stimulated microglia was found to be 590.64±160.72% of the level in untreated control (Fig. 4D). Pre-treatment with MgTx, PAP, or 4-AP prior to the addition of Tat significantly reduced the levels of ROS to 175.76±62.37%, 169.91±84.58%, and 252.70±89.4% of control, respectively. Collectively, these results strongly support a critical role for K_v_1.3 channels in the production and secretion of neurotoxins by Tat-activated microglia.

### Neurotoxic activity of Tat-stimulated microglia is mitigated by knockdown of K_V_1.3 gene

**Figure 5:**
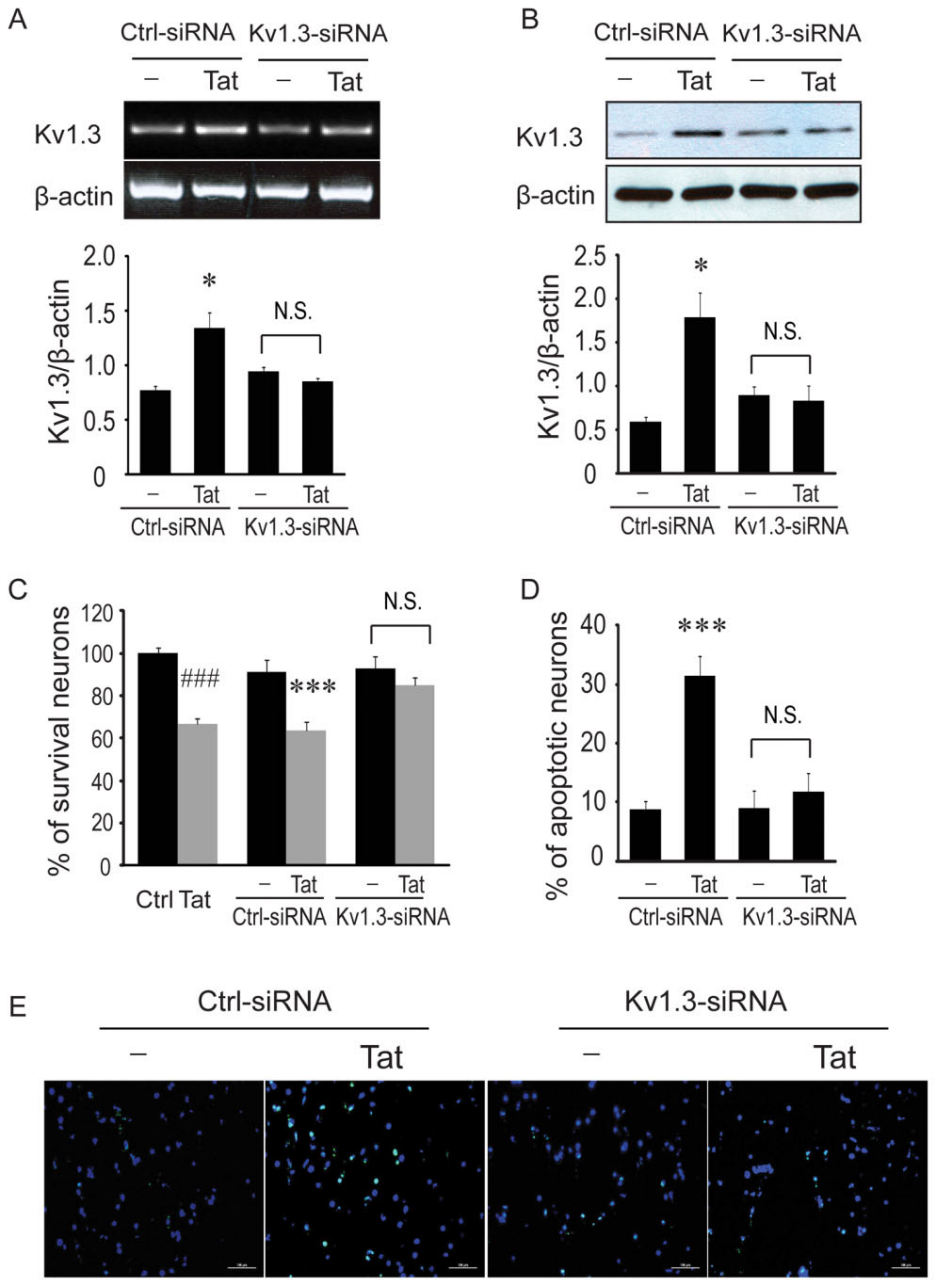
K_v_1.3 siRNA abrogates neurotoxic activity of Tat- activated microglia. Microglia were transfected with siRNA targeting K_v_1.3 (K_v_1.3-siRNA) or nonspecific GAPD control siRNA (Ctrl-siRNAS) for 48 or 72 hr, followed by an additional 24 hr exposure to Tat (200 ng/ml). Cells were then harvested for detections of K_v_1.3 mRNA (48 hr post-transfection/24 hr Tat treatment) and K_v_1.3 proteins (72 hr post-transfection/24 hr Tat treatment). Supernatants were subjected to neuronal culture. Neuronal apoptosis and viability assay were determined using TUNEL staining and MTT assay. **A**: Representative gels show RT-PCR products for K_v_1.3 mRNA and internal control β-actin and bar graph reflects the density of each band after normalization of its β-actin. **B**: Western blots show K_v_1.3 protein and internal control β-actin protein expression of microglia, and bar graph shows densitometric quantification of each band. **C**: Collected supernatants were subjected to primary neuronal culture at a dilution of 1∶5 for 24 hr and neuronal viability was evaluated by MTT assay. An increased viability was observed in neurons treated with supernatants recovered from microglia transfected with K_v_1.3-siRNA, but not transfected with Ctrl-siRNA. **D**: Transfection of microglia with K_v_1.3-siRNA significantly reduced neuronal apoptosis. In contrast, transfection of microglia with Ctrl-siRNA exhibited no significant protective effect. **E**: Apoptotic neurons were visualized by fluorescence microscopy at ×400 original magnification. Scale bar equals 100 µm. * p<0.05, *** p<0.001 vs Ctrl-siRNA; ^###^ p<0.001 vs Ctrl (blank).

Having demonstrated HIV-1 Tat upregulates K_v_1.3 expression in rat microglia and that K_v_1.3 channels are involved in Tat-induced microglia-mediated neurotoxicity, complementary experiments were next performed to address whether gene silencing by knockdown of the K_v_1.3 gene (KCNA3) with siRNA would attenuate these effects. First, microglia were transfected with K_v_1.3-siRNA or nonspecific GAPD control siRNA (control siRNA) for 48 hr or 72 hr, depending on whether mRNA or protein expression was to be measured, and incubated with or without 200 ng/ml Tat for an additional 24 hr. RT-PCR and western blot were then used to examine K_v_1.3 mRNA expression and K_v_1.3 protein levels, respectively. As expected, the upregulation of K_v_1.3 mRNA expression in Tat-stimulated microglia was efficiently inhibited by transfection with K_v_1.3 siRNA as compared to those transfected with control siRNA (Fig. 5A). Paralleling these results, Tat-enhanced K_v_1.3 protein expression was found to be significantly decreased with K_v_1.3 siRNA transfection (Fig. 5B).

To examine the effect of K_v_1.3 channel knockdown on the neurotoxic properties of Tat-exposed microglia, we again employed measures of neuronal viability and apoptosis. For this experiment, microglia were transfected with K_v_1.3-siRNA for 72 hr and then incubated with 200 ng/ml Tat protein an additional 24 hr. Microglial supernatants were next collected and applied at 1∶5 dilution to rat cortical neurons. Neurons were then incubated for an additional 24 hr before being assessed by MTT assay and TUNEL staining. As demonstrated by MTT assay, the decline in neuronal viability due to Tat-exposed microglial supernatants (66.87±2.55%) was improved by pre-transfection with K_v_1.3-siRNA (84.70±3.93%) (Fig. 5C). Similarly, TUNEL staining revealed the percentage of apoptotic neurons was decreased from 31.40±3.37% to 11.8±3.03% with K_v_1.3 gene knockdown (Fig. 5D & 5E). The capacity for K_v_1.3 gene knockdown to mitigate the neuronal damage caused by Tat-activated microglia indicates the upregulation of K_v_1.3 mRNA and protein is a key component in the mechanism of this neurotoxicity.

### ERK1/2 MAPK signaling pathway involvement in Tat-induced microglial neurotoxicity

**Figure 6:**
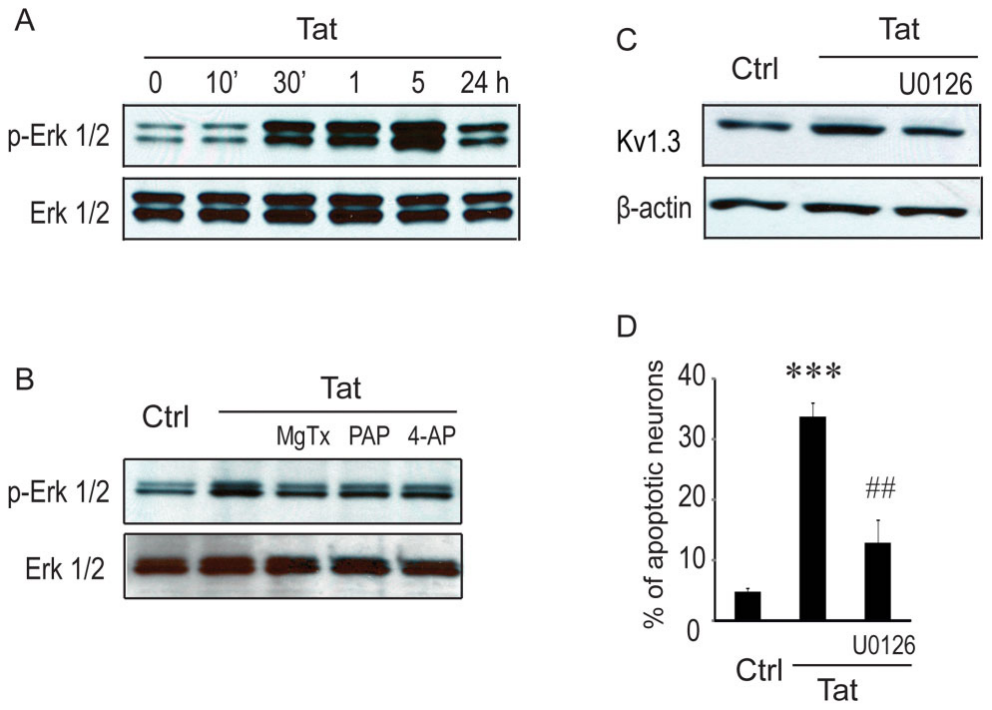
Involvement of ERK1/2 MAPK pathway in Tat-mediated upregulation of K_V_1.3 expression. **A**: Western blot analysis for ERK1/2 MAPK. Microglia were exposed to 200 ng/ml of Tat and harvested at indicated times. Protein expression was analyzed by immunoblot using antibodies against ERK1/2 phosphorylation (pERK1/2) and total ERK1/2 (ERK1/2) MAPK. Tat up-regulated pERK1/2 MAPK in a time window from 30 min to 5 hr. **B**: K_v_ channel antagonists inhibit ERK1/2 MAPK phosphorylation. Microglia were treated with MgTx (5 nM), PAP (10 nM), or 4-AP (1 mM) for 30 min followed by Tat at 200 ng/ml for additional 5 hr. Gel blots reveal a reduction of Tat-induced ERK1/2 phosphorylation in microglia treated with MgTx, PAP, or 4-AP, indicating a link between K_v_ 1.3 channel activation and ERK1/2 MAPK signal pathway. **C**: Western blot results showing that the blockade of Tat enhancement of K_v_1.3 expression in microglia was blocked by U0126, an inhibitor for MEK1 and MEK 2, further demonstrating the link between ERK1/2 MAPK and Tat-induced increase of K_v_1.3 expression. **D**: TUNEL staining exhibited a significant increase of neuronal apoptosis induced by the supernatants collected from Tat-treated microglia and its blockade by U0126, a MEK1 and MEK2 inhibitor. Data were from three independent experiments. *** *p*<0.001 vs Ctrl; ^##^ *p*<0.001 vs Tat-treated alone.

Thus far we have shown Tat-induced upregulation of K_v_1.3 channels and currents to be critical to the activation of microglia and subsequent damage to neurons. To better clarify the mechanisms underlying these observations, we next turned our attention to the extracellular signal-related kinases (ERK1/2) MAPK signaling pathway, which has been implicated elsewhere in chronic neurodegenerative disease and may mediate the channel profile alterations associated with Tat exposure [30], [31], [32]. For this experiment, microglia were exposed to 200 ng/ml Tat and proteins were harvested at various time points for assessment by western blot. Our results demonstrate ERK1/2 phosphorylation (pERK1/2) was enhanced by Tat exposure beginning at 30 min and peaking at 5 hr after stimulation (Fig. 6A). To determine if ERK1/2 activation mediates the Tat-induced increases in K_v_1.3 channel expression and subsequent neurotoxicity, U0126 was used to inhibit MEK1 and MEK2, the MAPK kinases responsible for phosphorylation of ERK1/2 MAPK. Reduction of ERK1/2 MAPK activity using U0126 (10 µM) was found to markedly reduce both the expression levels of microglial K_v_1.3 (Fig. 6C) and the neurotoxicity of supernatants collected from Tat-exposed microglia (Fig. 6D). These results provide evidence for the involvement of the ERK MAPK pathway in Tat-induced K_v_1.3 expression and consequent microglia neurotoxic activity. Lastly and perhaps surprisingly, application of MgTx (5 nM), PAP (10 nM), or 4-AP (1 mM) to microglia after 30 min of Tat exposure was also found to significantly inhibit the Tat-enhanced phosphorylation of ERK1/2 (Fig. 6B).

### K_V_1.3 channel involvement in ex vivo HIV-1 Tat-induced microglia-mediated neurotoxicity

**Figure 7:**
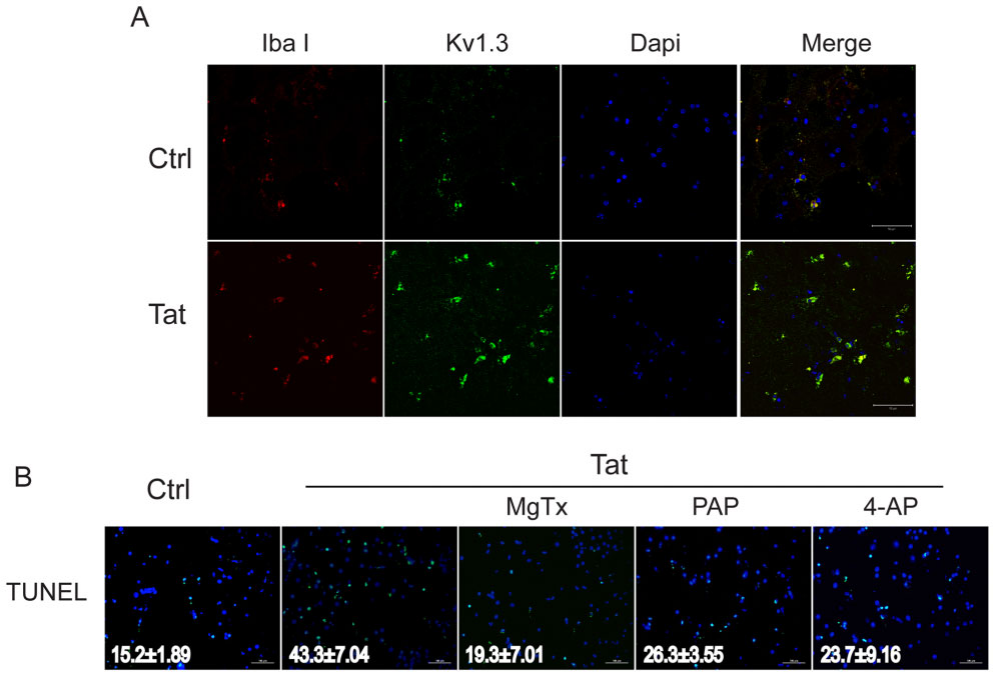
K_v_ channel blockers ameliorated Tat-induced microglia neurotoxicity in rat hippocampus slices. Rat hippocampus slices were pretreated with MgTx (5 nM), PAP (10 nM), or 4-AP (1 mM) for 30 min before addition of Tat (200 ng/ml). Immunohistochemistry or TUNEL staining was performed 24 hr later. **A**: Tat increased levels of K_v_1.3 expression that were co-localized with microglia (Iba1 staining) in rat hippocampus slices. Rat hippocampus slices were stained with mouse anti-Iba1 Ab (1∶1000, red), whereas K_v_1.3 was stained with goat polyclonal antibody (1∶200, green). Images were visualized by confocal microscopy. **B**: TUNEL staining showed that Tat produced neuronal apoptosis in rat hippocampus slices that was attenuated by MgTx, PAP, or 4-AP. Numerical numbers in each image panel represents the average apoptotic cells (M ± SD, n = 3 slices, 5 random visual fields were counted in each slice) in experimental conditions as indicated.

Thus far we have demonstrated Tat protein exposure results in the upregulation of K_v_1.3 expression and the production of neurotoxic substances in cultured microglia. To better approximate *in vivo* conditions, we next used a rat hippocampal slice culture to evaluate *ex vivo* the alterations in microglial K_v_1.3 expression and neuronal apoptosis resulting from HIV-1 Tat application. Hippocampal slices were first dissected from rats aged 20–30 days, cultured in NeuroBasal medium, and incubated for 24 hr with 200 ng/ml Tat protein. Brain slices were then double stained with the microglia marker Iba1 and K_v_1.3 antibodies. In Tat protein treated slices, K_v_1.3 expression was found to be enhanced and co-localized with Iba1 stained microglia (Fig. 7A). In addition, TUNEL staining revealed Tat-induced neuronal apoptosis could be attenuated with 30 min K_V_ channel antagonist pre-treatment, either MgTx (5 nM), PAP (10 nM), or 4-AP (1 mM) (Fig. 7B). These results are fully consistent with our *in vitro* studies and corroborate the involvement of K_v_1.3 channels in the neuronal damage caused by Tat-activated microglia.

## Discussion

Microglia are functionally related to cells of the monocyte/macrophage lineage and play an important role as resident immunocompetent phagocytic cells in the HAND pathogenesis. A prominent pathological feature in HIV-1-infected brain is microglia activation and the activated microglia exert neurotoxic effects in the brain by releasing a variety of potentially neurotoxic substances. In addition to their production of neurotoxins, microglia express a large number of chemokine receptors that are involved in cell migration and serve as co-receptors for HIV-1 infection. Indeed, microglia are the predominant resident CNS cell type productively infected by HIV-1 [8], [9]. Due to poor penetration of antiretroviral drugs through the blood-brain barrier (BBB), resident microglia (and brain macrophages) constitute a cellular reservoir of HIV-1 in the brain and a source of potential neurotoxic substances [33], [34], [35]. Thus, suppression of microglia production of neurotoxins is critical to the control of HAND onset and progression. In the present study, we demonstrated HIV-1 Tat-induced microglia activation and associated neurotoxicity requires K_v_1.3 channel activity. Exposure of microglia to HIV-1 Tat protein was found to enhance both K_v_1.3 currents and production of neurotoxins including IL-1β, TNF-α, NO and ROS, ultimately leading to neuronal damage. Suppression of K_v_1.3 channels, either by K_v_1.3 channel antagonists or via gene knockdown, significantly inhibited Tat-induced microglia-mediated neurotoxicity. These findings indicate K_v_1.3 channel modulation has the potential to mitigate microglia-associated neurotoxic activity.

Microglia express a defined pattern of K_v_ channels including inward rectifier K_ir_2.1 and outward rectifiers K_v_1.5 and K_v_1.3 [19], [36]. Exposure to a variety of stimuli produces a characteristic pattern of up-regulation of K_v_1.3 and activation of microglia results in neuronal injury via a process requiring K_v_1.3 activity [13], [14], [15], [16]. In a previous study we found HIV-1 gp120 exposure activates microglia, in conjunction with enhanced K_v_1.3 expression and outward K^+^ currents, leading to neuronal apoptosis [24], [25]. The gp120-induced microglial neurotoxicity was significantly attenuated via suppression of K_v_1.3 expression or blockade of K_v_1.3 current. Similarly, our present study revealed exposure to HIV-1 Tat increases microglial K_v_1.3 channel expression and outward K^+^ current in association with activation, neurotoxin secretion, and neuronal apoptosis, which were successfully inhibited by either siRNA knockdown of the K_v_1.3 gene (Fig. 4) or specific K_v_1.3 blockers MgTx and PAP (Fig. 1, Fig. 2, and Fig. 7). We confirmed these results in an *ex vivo* study using hippocampal slice culture, in which HIV-1 Tat-induced microglia-mediated neuronal apoptosis was attenuated by pre-treatment with K_v_1.3 antagonists MgTx or PAP, or a broad spectrum Kv channel blocker, 4-AP. Collectively, these findings reveal the integral role of K_v_1.3 channels in regulating microglia activation and establish a new approach for controlling microglia mediated neurotoxic activity.

Although reported in several studies to be associated with diseases including B cell lymphoma [37], breast cancer [38], [39] and Alzheimer's disease [40], research on the importance of enhanced microglial K_v_1.3 channel activity in HIV-1 related cognitive impairment has thus far been sparse. The evidence for the pivotal role of microglia in HAND pathogenesis is abundant however, as microglia are well known to mediate HIV entry into the brain, serve as a reservoir for productive and latent HIV-1 infection, and function as a source of neurotoxic substances [8], [19], [41], [42], [43]. After infection with HIV-1, microglia undergo dramatic phenotypic, immunological, and functional changes to produce the cytokines, chemokines, superoxides, and viral proteins that result in neuronal injury. In order to determine the role of microglial K_v_1.3 in this process, we examined the neurotoxic secretions of HIV-1 Tat-treated microglia in relation to K_v_1.3 channel activity. We found blockade of K_v_1.3 channels using either specific K_v_1.3 antagonists, MgTx and PAP, or a broad spectrum K_V_ blocker, 4-AP, was sufficient to inhibit microglial production of IL-1β, TNF-α, NO, and ROS. These results are consistent with our previous findings [24] and suggest enhanced microglial K_v_1.3 channel activity is required for the HIV-1 Tat-induced secretion of neurotoxins by microglia.

HIV-1 Tat exposure has been shown to lead to microglia/macrophage activation, neurotoxin secretion, and subsequent neuronal damage [22], [44], [45], [46] in a process mediated through microglial signal transduction pathways such as ERK1/2, PI3K, and p38 MAPK [47], [48]. Tat protein has also been shown capable of increasing an outward-rectifying K^+^ current in rat microglia through regulation of transcription factor NF-κB [49]. However, while K_v_1.3 channel activity has here been demonstrated to be necessary for Tat-induced microglial neurotoxicity, the underlying connection remains to be elucidated. Although the breadth of mechanisms for modulating K_v_ channels are numerous, including regulation of gene expression, post-translational modification, direct interactions with organic molecules and peptides, and responsiveness to membrane potential, to name a few, we chose as an appropriate starting place those signaling pathways which can convert an extracellular signal such as HIV-1 Tat protein into a functional cellular response. In particular, we focused on ERK1/2, which constitute one of the MAPK pathways that commonly transduce microenvironmental conditions in microglia and have been implicated in chronic neurodegenerative diseases [30], [31], [32]. Depending on the cell type, the stimulus, and the duration of cell activation, a variety of biological responses including cell proliferation, differentiation, migration, and apoptosis have been correlated with ERK activation [30], [31], [32]. In the present study, we explored whether ERK1/2 activation was involved in the enhancement of microglial K_v_1.3 expression and neuronal apoptosis resulting from exposure to HIV-1 Tat. We found ERK1/2 phosphorylation increased in HIV-1 Tat-treated microglia in a time-dependent manner (Fig. 6A), but could be prevented by pre-treatment with U1026, an inhibitor of the upstream kinase responsible for regulating ERK1/2 activity. Furthermore, pre-treatment with U1026 ameliorated Tat-induced microglial K_v_1.3 expression and associated neurotoxicity (Fig. 6C, Fig. 6D), indicating this process is dependent on activation of the ERK1/2 MAPK pathway. Lastly, our experiments revealed the novel finding that HIV-1 Tat-induced ERK1/2 phosphorylation could be inhibited with K_v_ channel antagonists, MgTx, PAP, or 4-AP. It appears that while Tat-induced microglial K_v_1.3 expression is dependent on ERK1/2 MAPK, this same pathway is also responsive to K_v_ currents. Given that K_v_ currents set the membrane potential and thus influence Ca^2+^ influx, the latter effect may be mediated through Ca^2+^-dependent intracellular processes or pathways. While more investigation is necessary, this reciprocal regulation may allow for intervention in the crucial transition between functional immune activation and reactive microgliosis.

In this study, we provided evidence demonstrating K_v_1.3 channels to be an integral component of HIV-1 Tat-induced microglia-mediated neurotoxicity and a potential site of regulation. This promising data opens the future possibility of using K_v_1.3 channel inhibitors as a novel strategy to combat HAND and other neurodegenerative disorders in which the pathophysiological process involves microglia-mediated immune and inflammatory responses. In a previous *in vivo* study, we demonstrated the administration of K_v_ channel antagonist 4-AP could ameliorate HIV-1-induced encephalitis and cognitive disorder, improving spatial learning and memory in a severe combined immunodeficient (SCID) mouse model of HIV-1 encephalitis (HIVE) [50]. Nevertheless, as K_v_ channel blockers selectively target immune cells including macrophage, microglia, and lymphocytes, the therapeutic benefit of this approach must be carefully considered for potential risks to the immune system. Of these cells, the most abundant K_v_1.3 channel expression is reported to be found the human effector memory T cells (CD4^+^CCR7^−^CD45RA^−^), which regulate Th1 cell inflammatory responses [36], [51], [52]. The use of small molecule K_V_ blockers, such as verapamil, dilitazem, and nifedipine, has been shown to reduce IL-12 secretion and inhibit T cell proliferation [53]. Notably, these immunomodulatory effects have been found to depend heavily on the level of K_v_1.3 channel expression, which changes dramatically as T cells differentiate from naïve to memory states or transition from resting to activation [54], [55], [56]. Given that the immune response functions of microglia, macrophage, Helper T cells, and B cells remain basically intact, the potential side effects of pharmacologically blocking K_v_1.3 channels could be minimal [52], [57]. In fact, the potential side effects of using K_V_ channel blockers to treat a wide range of autoimmune conditions, including those involving effector memory T cells, delayed type hypersensitivity, type 1 diabetes, rheumatoid arthritis, multiple sclerosis, and inflammatory bone resorption, have been investigated without revealing any generalized immunesuppression [58], [59], [60], [61]. Recently, the safety of this approach was given further credence when the US Food and Drug Administration approved the K_V_ channel blocker dalfampridine (Ampyra) as a treatment multiple sclerosis (http://www.fad.gov/NewsEvents/Newsroom/PressAnouncements/ucm198463.htm).

In summary, the present study serves to establish the integral role of K_v_1.3 channel activity in HIV-1 Tat-induced microglia-mediated neurotoxicity. The identification of K_v_1.3 channels as a point of intervention in this process may open new avenues for therapeutic modalities. Given its feasibility and safety, it may now be advantageous to consider studying a K_v_1.3 channel-based therapeutic approach in the treatment of HAND and other neurodegenerative disorders characterized by microglia-mediated neuroinflammation.
