# Correction: HIV-1 Tat Protein Increases Microglial Outward K^+^ Current and Resultant Neurotoxic Activity

**DOI:** 10.1371/journal.pone.0109218

**Published:** 2014-09-19

**Authors:** The PLOS ONE Staff

There are errors in Figure 5 (E) of the published article. The authors have provided a corrected Figure 5 here.

**Figure 5:**
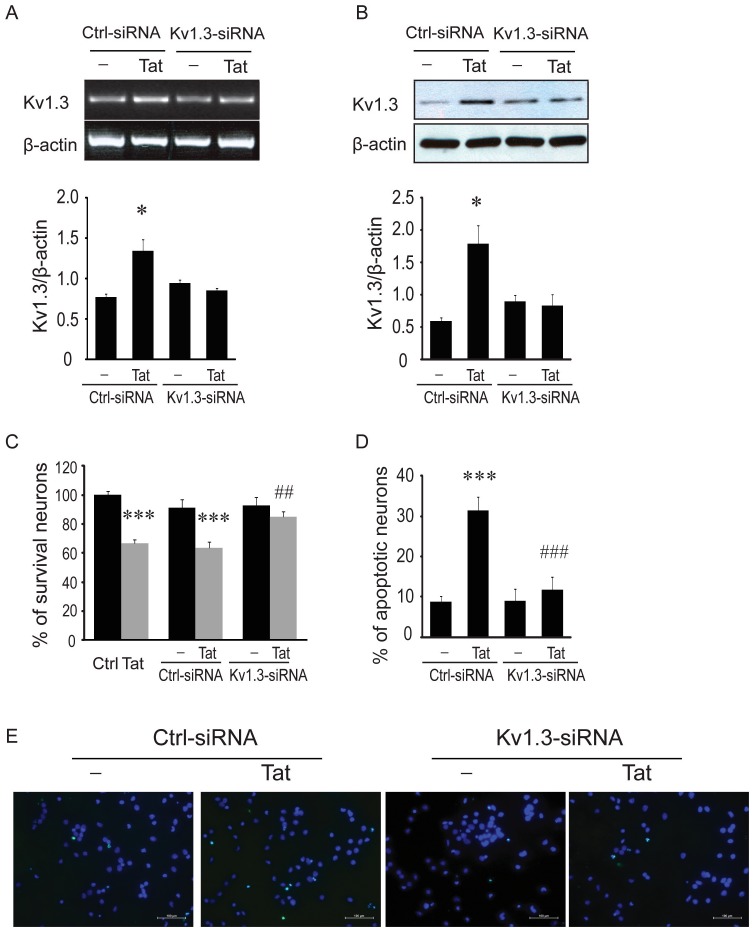
K_v_1.3 siRNA abrogates neurotoxic activity of Tat- activated microglia. Microglia were transfected with siRNA targeting K_v_1.3 (K_v_1.3-siRNA) or nonspecific GAPD control siRNA (Ctrl-siRNAS) for 48 or 72 hr, followed by an additional 24 hr exposure to Tat (200 ng/ml). Cells were then harvested for detections of K_v_1.3 mRNA (48 hr post-transfection/24 hr Tat treatment) and K_v_1.3 proteins (72 hr post-transfection/24 hr Tat treatment). Supernatants were subjected to neuronal culture. Neuronal apoptosis and viability assay were determined using TUNEL staining and MTT assay. A: Representative gels show RT-PCR products for K_v_1.3 mRNA and internal control β-actin and bar graph reflects the density of each band after normalization of its β-actin. B: Western blots show K_v_1.3 protein and internal control β-actin protein expression of microglia, and bar graph shows densitometric quantification of each band. C: Collected supernatants were subjected to primary neuronal culture at a dilution of 1:5 for 24 hr and neuronal viability was evaluated by MTT assay. An increased viability was observed in neurons treated with supernatants recovered from microglia transfected with K_v_1.3-siRNA, but not transfected with Ctrl-siRNA. D: Transfection of microglia with K_v_1.3-siRNA significantly reduced neuronal apoptosis. In contrast, transfection of microglia with Ctrl-siRNA exhibited no significant protective effect. E: Apoptotic neurons were visualized by fluorescence microscopy at ×400 original magnification. Scale bar equals 100 µm. * p<0.05, *** p<0.001 vs Ctrl-siRNA; ^###^ p<0.001 vs Ctrl (blank).
